# Renal denervation is effective in reducing blood pressure in patients with CKD

**DOI:** 10.1093/ckj/sfaf126

**Published:** 2025-06-04

**Authors:** Agnes Bosch, Dennis Kannenkeril, Roland E Schmieder

**Affiliations:** Department of Nephrology and Hypertension, Friedrich-Alexander-University Erlangen-Nürnberg (FAU), Erlangen, Germany; Department of Nephrology and Hypertension, Friedrich-Alexander-University Erlangen-Nürnberg (FAU), Erlangen, Germany; Department of Nephrology and Hypertension, Friedrich-Alexander-University Erlangen-Nürnberg (FAU), Erlangen, Germany

**Keywords:** blood pressure, chronic kidney disease, chronic renal insufficiency, renal denervation, renal function

## Abstract

Hypertension is a major cause and the predominant accelerator of progressive loss of renal function in patients with chronic kidney disease (CKD). Despite advances in pharmacological intervention in recent years, a significant proportion of patients with CKD have uncontrolled, often treatment-resistant hypertension, necessitating alternative therapeutic approaches to control hypertension and slow the progression of renal function decline. Renal denervation modifies efferent and afferent renal sympathetic nerve activity and thus addresses an important modifier of both, blood pressure and renal function that has not been adequately addressed by pharmacologic therapies. This article reviews the current evidence on renal denervation in hypertensive patients with CKD. Safety and efficacy data from clinical trials and observational studies are reassuring that renal denervation is emerging as a promising additional treatment option for patients with uncontrolled hypertension and CKD. However, further randomized controlled data are needed to support these findings, particularly in patients with advanced CKD.

## INTRODUCTION

Arterial hypertension is one of the most prevalent cardiovascular risk factors worldwide, yet treatment rates remain suboptimal in many regions of the world [1]. Even in the Western Hemisphere only about 40% of individuals with hypertension are treated and controlled to less than 140/90 mmHg [2]. This is a staggering situation, given that hypertension is the most influential modifiable risk factor for all-cause and cardiovascular disease morbidity and mortality worldwide, surpassing other prominent factors such as body mass index and non-high-density lipoprotein cholesterol [3]. Hypertension is also a major cause and predominant accelerator of the progressive loss of renal function [4]. Despite advances in pharmacological intervention in recent years, a significant proportion of patients with CKD have uncontrolled, often treatment-resistant hypertension, necessitating alternative therapeutic approaches to control hypertension, reduce associated cardiovascular disease and slow the progression of renal function decline [5, 6].

## EFFICACY OF RDN IN THE GENERAL HYPERTENSIVE POPULATION

Renal denervation (RDN) modulates efferent and afferent renal sympathetic nerve activity resulting in a reduction in blood pressure (BP) [7]. RDN now has a 20-year history of clinical development. The first open-label studies suggesting a pronounced BP-lowering effect of RDN in patients with severe treatment-resistant hypertension [8] were followed by the first generation of randomized sham-controlled trials. The (Renal Denervation in Patients With Uncontrolled Hypertension (SYMPLICITY HTN-3)) trial failed to provide proof-of-concept for RDN, with no statistically significant difference in BP reduction in the RDN group compared with the sham group was found. Subsequent analysis of the SYMPLICITY HTN 3 trial identified several confounding factors that explained these results, such as incomplete denervation, drug turbulence during the run-in and follow-up periods, and racial/socioeconomic factors [9–11] In the meantime, the procedural and methodological confounders identified in the first generation of clinical trials have been addressed and second-generation sham-controlled trials have been conducted. For example, renal nerve density was shown to be highest in the post-bifurcation region; 94% of nerves were found to be located within 3 mm of the renal artery lumen post-bifurcation compared with 46% in the proximal segment [12]. In addition, it was found that renal nerves may not fully converge until after the main bifurcation [13].

**Figure 1:**
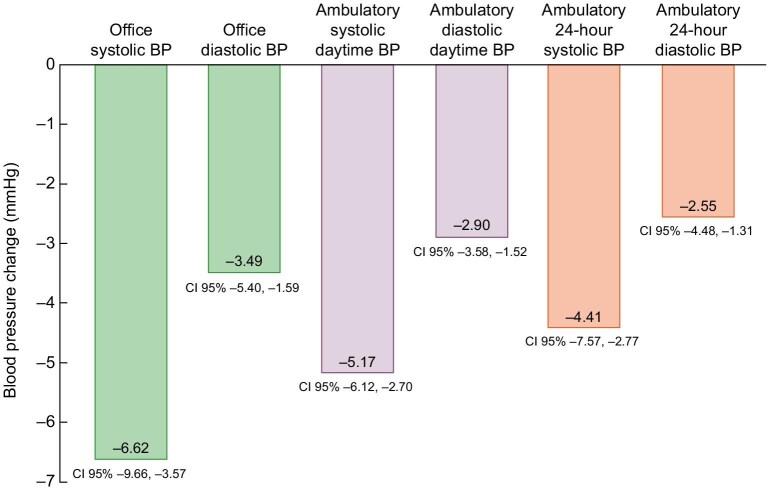
Sham corrected blood pressure lowering efficacy of renal denervation in 2478 patients from 13 clinical trials with median follow-up duration of 3 months.

Data on the long-term efficacy of RDN include real-world evidence showing sustained BP reductions and reduced medication burden after RDN for up to 9 years [14–17]. A meta-analysis included 18 reports and more than 4400 patients [18]. RDN has been shown to be effective in lowering BP in a variety of patient subgroups [19]. A meta-analysis of 2478 patients with hypertension who were either off or on treatment examined the BP-lowering efficacy of radiofrequency-based and ultrasound-based RDN with a median follow-up to the primary endpoint of 3 months (ranging from 2 to 6 months across trials) [18]. The outstanding strength of this meta-analysis among all the other published meta-analyses is the inclusion criteria of using only sham-controlled randomized prospective trials, thereby increasing the scientific evidence of the results. Figure 1 shows the reduction in office and 24-h/daytime BP. Of note, the BP changes between the renal denervation and sham groups were similar in the trials with and without concomitant antihypertensive medication.

**Figure 2:**
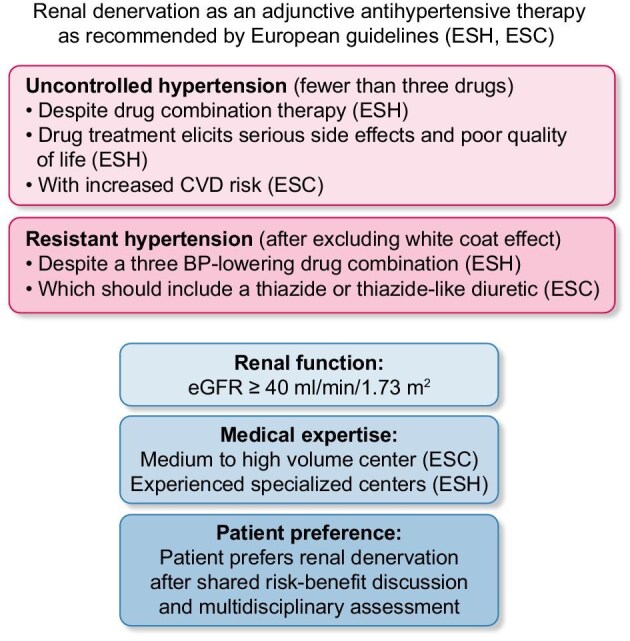
Renal Denervation as an adjunctive antihypertensive therapy as recommended by European guidelines (ESH, ESC).

Both, the European Society of Cardiology (ESC) Guidelines for the Treatment of Hypertension 2024 and the European Society of Hypertension (ESH) Guidelines 2023 state as Class II recommendations that: “Several randomized controlled clinical trials have demonstrated that renal denervation is a safe procedure that effectively reduces blood pressure in patients with arterial hypertension” [20, 21]. Figure 2 summarizes the ESC and ESH guideline recommendations for RDN, which are basically consistent despite some differences in wording and focus on different aspects related to their society structure. Importantly, the ESH and ESC guidelines state that due to lack of evidence renal denervation is not recommended in patients with an estimated glomerular filtration rate (eGFR) <40 mL/min/1.73 m² unless further evidence becomes available [20, 21]. Now, in 2025, what is the evidence that RDN may or should be used in hypertensive patients with reduced renal function across the CKD stages?

## RATIONALE FOR RDN IN CKD PATIENTS

Patients with CKD are at very high cardiovascular risk and exhibit increased sympathetic nervous system (SNS) activity [22, 23]. Increased efferent SNS to the kidneys leads to activation of the renin–angiotensin system (RAS), sodium retention and decreased renal perfusion, thereby exaggerating hypertension, further impairing renal function and adversely affecting cardiovascular prognosis [7, 24, 25]. Experimental studies have shown that SNS activity increases significantly with progression of CKD [7, 25, 26]. Patients with end-stage renal disease (ESRD) on hemodialysis showed, as an anatomical substrate, a significant increase in nerve endings in the internal area of the renal artery adventitia compared with patients with normal renal function or less severe CKD [27]. A concise analysis of several clinical studies, all using microneurography, showed that GFR in patients with CKD was inversely correlated with SNS activity [28].

**Figure 3:**
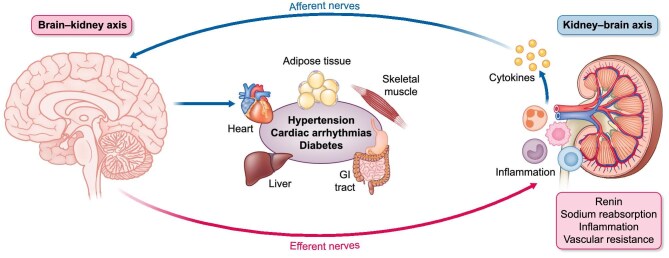
Schematic overview of the bidirectional kidney-brain axis with relevant efferent and afferent pathways.

In addition to the efferent SNS activity to the kidneys, afferent neural signaling from the failing native kidney to the brain is also considered a key mediator in the process of increased central SNS activity, which in turn affects the entire cardiovascular system and worsens cardiovascular and renal prognosis [24]. In patients with ESRD and chronic hemodialysis, bilateral nephrectomy reduced increased SNS activity as evidenced by microneurography, decreased systemic arterial pressure and decreased peripheral vascular resistance [26]. This suggests that afferent sensory nerve signalling from the diseased kidneys to the central SNS is an important pathophysiological mechanism in CKD leading to SNS overactivity and hypertension. The bidirectional kidney–brain axis with relevant efferent and afferent pathways mediating this vicious cycle between increased SNS activity, CKD and hypertension is shown in Fig. 3 [29].

Comprehensive management of patients with CKD involves many targets, but none of the classic and recently recommended treatment approaches, such as sodium-glucose linked transporter 2 inhibitors, glucagon-like peptide-1 receptor agonists and non-steroidal mineralocorticoid receptor antagonists, targets the SNS [30]. RDN targets elevated BP and increased SNS activity, both of which have been identified as major progression factors in CKD. Thus, RDN may be a promising adjunctive treatment option in this patient population. As a condition sine qua non, the safety of RDN is of great importance, especially in these hypertensive patients with CKD. What are the safety concerns of the RDN procedure that need to be addressed in patients with CKD?

## SAFETY OF RDN IN PATIENTS WITH CKD

Clinical trials have demonstrated the overall procedural safety of RDN in various hypertensive patient cohorts over the past 15 years by showing no difference in major adverse events with RDN compared with control groups [31–37]. These findings reassure the use of RDN in the general hypertensive population, with the limitation that follow-up data in larger study cohorts (from clinical trials and observational studies) are only available up to 3 years [38]. Importantly, the clinical trials included patients with moderate CKD, but excluded those with advanced CKD. Safety data in patients with an eGFR <45 mL/min/1.73 m^2^ are limited to mostly smaller studies.

Potential procedural complications of concern to patients and physicians include contrast-induced nephropathy, long-term eGFR loss, vascular injury, and *de novo* renal artery stenosis. These potential complications are discussed in detail below.

### Procedure-related complications

A meta-analysis of procedure-related events in 238 patients with CKD from 11 single-center, non-randomized, uncontrolled studies found no serious procedure-related complications (including all-cause death, major cardiovascular events, perioperative complications and hypertensive crisis) [39]. Adverse reactions related to puncture site complications, which are common in percutaneous angiographic procedures, included one case of bleeding, three cases of pseudoaneurysm (1.3%) and seven cases of hematoma (2.9%). No incidence of electrolyte disturbances, including hyperkalaemia, hypokalaemia, hypercalcemia or hypocalcaemia was observed [39].

### De novo renal artery stenosis

Patients with uncontrolled hypertension, who often have other concomitant cardiovascular risk factors, have a naturally increased incidence of renal artery stenosis. The prevalence of arteriosclerotic renal artery stenosis identified by Doppler ultrasound in more than 1 million US citizens ranged from 0.5% to 7% of individuals [40].

In a meta-analysis of 5769 patients in 50 clinical trials the annular incidence of renal artery stenting after radiofrequency RDN was estimated to be 0.2%, which is comparable to the natural increased incidence of renal artery stenosis [41]. In other words, we have no evidence that the RDN procedure is associated with new development of renal artery stenosis. This is further confirmed by a most recent analysis based on imaging techniques (duplex ultrasound, computerized tomography angiography and/or magnetic resonance angiography). No association with vascular injury or induction of RAS was observed during 36 months of follow-up in 557 patients [42].

In patients with advanced CKD, atrophic renal arteries are common [43]. A case series of ESRD treatment with RDN emphasizes this, as RDN was only possible in three-quarters of the enrolled patients due to very small renal artery diameters [24]. Second-generation RDN catheters, with confirmed efficacy in sham-controlled trials, allow treatment of renal arteries with an internal diameter of 3–8 mm [44]. Accessory vessels should be treated if they fall within the required diameter ranges [44]. Is there a higher risk of renal artery stenosis after RDN in patients with CKD? Patients with CKD were analyzed separately as a subset of the above meta-analysis. No adverse events in the distal arteries were found in this group of 396 patients [41]. The retrospective analysis of 3-year follow-up data from the Global Symplicity Registry also showed no difference in safety outcomes between subjects with and without CKD and found rates of renal artery stenosis <0.5% [45].

### Decline in eGFR

Long-term data clearly show that RDN does not affect long-term renal function including eGFR and albuminuria [19, 45]. Further details on long-term data on eGFR in patients with CKD separated by degree of renal function impairment are provided below.

Data on short-term changes in eGFR in patients with CKD after RDN are limited. Given the required use of iodinated contrast media during RDN a possible short-term adverse effect of RDN on renal function (acute kidney injury) needs to be discussed. A single-center observational study in 40 patients with treatment-resistant hypertension reported a transient increase in serum creatinine, defined as absolute increase of approximately 0.3 mg/dL from basal values in three patients (13.4%) in the eGFR <45 mL/min group and one patient (5.3%) in the other group [46]. The transient increase in serum creatinine normalized within 48 h after hydration therapy [46]. Similarly, another study reported no difference in eGFR and urine albumin creatinine ratio (UACR) 1 month after RDN in 30 patients with CKD 2–4 compared with the values before RDN [47]. A recent meta-analysis of randomized controlled clinical trials including 2478 patients with various degrees of hypertension and patients with mild to moderate renal impairment reported no significant difference in the predefined safety outcomes between RDN and sham groups, including new-onset of ESRD, but short-term changes in renal function were not reported separately in this analysis [18].

It is known from other contrast-enhanced procedures that the amount of contrast media used and the degree of renal impairment determine the likelihood of contrast-induced acute kidney injury. Adequate peri-procedural patient hydration is generally recommended [44]. In patients with CKD, adequate volume therapy may be difficult and must take into account cardiac function, especially in patients with impaired residual diuresis. Contrast media can be diluted to reduce the amount used [44]. A total contrast volume limit of less than two times the patient’s eGFR is recommended [48]. It is also important to consider that the amount of contrast media required in clinical trials may be higher than in the real-world RDN setting due to the additional angiographic documentation required in clinical trials. In addition, alternative contrast agents, such as carbon dioxide, may be considered [44].

### Vascular damage in patients with CKD

When comparing radiofrequency and ultrasound technology different approaches have been used to prevent vascular injury: radiofrequency-based RDN uses blood flow for cooling to protect the endothelial layer and energy is not delivered in a flat 360-degree plane with the SPYRAL system [49]. If there is overheating of the vessel wall (measured directly), there is an immediate cessation of radiofrequency energy delivery [44]. In ultrasound-based RDN, energy is delivered in a 360-degree plane and a saline-filled balloon is used for cooling, preventing any vascular injury. Both systems have been extensively analyzed in animal studies and have been found not to harm the vessel wall. Clinically, there was no difference in safety signals between the two Food and Drug Administration (FDA)-approved systems [49].

**Figure 4:**
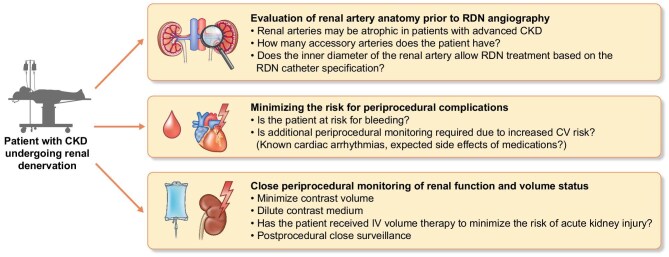
Practical periprocedural suggestions for patients with CKD undergoing renal denervation.

General periprocedural recommendations have been summarized previously [44]. Some practical periprocedural considerations, including lessons learned from treating CKD patients enrolled in the Erlangen Registry [50] and the Global Symplicity Registry [45] are summarized in Fig. 4.

## RDN IN PATIENTS WITH CKD STAGE 3 TO 4

The data from clinical studies on RDN investigating the BP-lowering efficacy and kidney function in patients with CKD stage 3 to 4 and patients with CKD stage 5 on hemodialysis are summarized in Table 1.

**Table 1:** Summary of RDN data on efficacy and kidney function in patients with CKD stage 3 to 5 and patients on hemodialysis.

Study characteristics, year of publication	Patients’ characteristics	BP-lowering efficacy	Kidney function
Global Symplicity Registry [45] (2022)	475 patients with eGFR <60 mL/min/1.73 m^2^, 1505 patients with eGFR ≥60 mL/min/1.73 m^2^	Reduction in BP (between baseline and 36 months) was independent of renal function	eGFR decline per year not significantly different between the CKD and non-CKD groups 1 year after RDN
Global SYMPLICITY Registry DEFINE [51] (2024)	864 patients, CKD stage 3–5	Office systolic BP, office diastolic BP and 24-h ambulatory diastolic BP were significantly lower at 3 years post-RDN procedure compared with baseline in all eGFR groups	After initial changes from baseline to 6 months that were related to the regression to the mean, the observed decline per year was similar to the age-dependent decline per year
Erlanger Registry [50] (2024)	47 patients with and 127 without CKD stage 3 or 4	Similar BP reduction in patients with and without CKD at any time point up to 12 months	12 months after RDN no significant decline of eGFR compared with the baseline value in both groups
Meta-analysis by Xia *et al*. [39] (2021)	11 single-center studies, 238 patients, CKD 2–5	Office BP and 24-h ambulatory BP (24 h-ABP) with significant reduction 1 month after RDN and 24 months after RDN	eGFR measurements after RDN were not significantly different from those obtained before RDN, UACR levels were significantly reduced at 3 months and 6 months after RDN
Randomized, sham controlled, multicentre, clinical trial, RDN-CKD, expected (2025)	20 patients with baseline and 6 months follow-up	RDN group: significantly lower 24-h ambulatory diastolic BP and office diastolic BP, no significant changes in 24-h ambulatory systolic BP compared with sham	Change in eGFR did not change significantly from baseline in either group, UACR was not different between groups at 6 months
Propensity score matching, RADIANCE [52] (2024)	Patients with mild CKD stage 3 (eGFR 40–60 mL/min/1.73 m^2^),	RDN reduced BP to the same extent in both CKD and non-CKD patients	eGFR not adversely affected
Observational study, Ott *et al*. [53] (2015)	27 patients with uncontrolled resistant hypertension and stage 3 CKD (eGFR 30–59 mL/min/1.73 m^2^)	Significant reduction in office BP and average 24-h ambulatory BP 1 year after RDN	Stabilization of eGFR 12 months after RDN compared with the progressive eGFR decline during the 3 years prior to RDN, significant reduction in albuminuria after RDN in patients with elevated UACR
Observational study, Hering *et al*. [54] (2012)	15 patients, CKD stage 3–4 (mean eGFR, 31 mL/min per 1.73 m^2^)	Office systolic and diastolic BP decreased at 1, 3, 6 and 12 months post-RDN, night-time ambulatory BP significantly decreased	Favorable short-term safety profile during first 12 months after RDN
Observational study, Hering *et al*. [55] (2017)	46 patients with stage 3 CKD	RDN significantly reduced 24-h daytime systolic BP from baseline to 24 months post-procedure	Stabilization of eGFR for up to 24 months after RDN
Observational study, Kiuchi *et al*. [47] (2016)	30 patients, CKD stage 2 to 4	significant reduction in office BP and 24-h ambulatory BP between baseline and 24-month follow-up	eGFR increased from baseline after RDN, the difference remained significant at all time points through the end of follow-up at 24 months
Observational study, Schlaich *et al*. [24] (2013)	ESRD, 9 patients treated with RDN, 3 untreated patients	Compared with baseline, office systolic BP was significantly reduced at 3, 6 and 12 months after RDN in the treated patients, whereas no change was observed in the untreated patients	Sympathetic nerve activity was substantially reduced in 2 patients who underwent repeat assessment
Case series, Ott *et al*. [59] (2019)	ESRD, 6 patients treated with RDN	Significant reduction in 24-h ambulatory BP 6 months after RDN, no change in antihypertensive medication	No change in hemodialysis parameters during follow-up
Case series, Scalise *et al*. [60] (2020)	ESRD, long-term hemodialysis, treatment-resistant hypertension, 12 patients	Office systolic and diastolic BP and 24-h ambulatory BP showed early and sustained reductions 1 and 12 months after RDN, no change in average number of antihypertensive medication	No significant periprocedural complications

### BP-lowering efficacy in clinical studies

Analysis of 3-year follow-up data from the Global Symplicity Registry showed that the reduction in BP achieved after RDN is independent of renal function [45]. In subjects with data on both baseline and 36-month BP data, the magnitude of office and 24-h ambulatory BP reduction was similar in patients with CKD (baseline eGFR <60 mL/min/1.73 m^2^ but ≥15 mL/min/1.73 m^2^, 475 patients) compared with patients without CKD (baseline eGFR ≥60 mL/min/1.73 m^2^, 1505 patients) 36 months after RDN [45].

Further evidence of similar BP-lowering efficacy in patients with CKD compared with those without CKD comes from the Erlanger Registry, a single-center experience, which showed similar reductions in 24-h, daytime and nighttime ambulatory, and office BP in patients with and without CKD at any time point up to 12 months [50]. There is very limited experience with RDN treatment in patients with CKD 3 and 4. A meta-analysis of 11 single-center studies including 238 patients with CKD 2–5 showed a sustained reduction in office and 24-h ambulatory BP between baseline and 24 months after RDN [39]. A most recent analysis of patients treated with RDN in the Global SYMPLICITY Registry DEFINE included 2540 patients without CKD, 498 patients with CKD stage 3a and 366 with CKD stage 3b or 4, categorized according to their baseline eGFR. Office and 24-h ambulatory BP showed clinically meaningful and comparable reductions from baseline to 12 months after RDN in patients with moderate to severe CKD, with no safety signals observed in patients with CKD [51].

### Efficacy in lowering BP in sham-controlled studies

**Figure 5:**
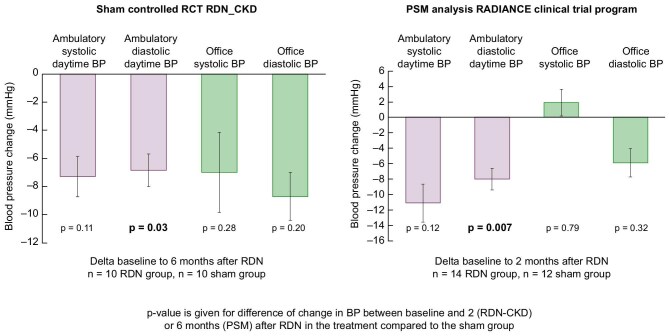
Sham corrected change in daytime systolic and diastolic BP after RDN treatment (X+SEM) in the sham controlled RCT (RDN-CKD) left side, and in the PSM analysis of the sham controlled RADIANCE Clinical Trial Program right side.

In a multicenter, prospective, sham-controlled, randomized, blinded, investigator-initiated study, the RDN-CKD trial (Effect of renal denervation on BP in patients with chronic kidney disease and uncontrolled hypertension, clingov: NCT04264403), the BP lowering effect was analyzed in patients with CKD stages 3a and 3b. The study was stopped early due to recruitment difficulties (COVID-19 pandemic). Patients treated with RDN had a significantly lower 24-h ambulatory diastolic BP and office diastolic BP compared with the sham intervention. No significant changes in 24-h ambulatory systolic BP were seen, most likely due to the small number of patients included (Fig. 5 left side). A propensity score matching analysis of the RADIANCE clinical trial program using ultrasound-based RDN in patients with mild CKD stage 3 (eGFR 40–60 mL/min/1.73 m^2^) showed that ultrasound-based RDN reduced BP to the same extent in both CKD and non-CKD patients. Consistent with this, the reduction in diastolic and systolic BP in patients treated with ultrasound-based RDN compared with the sham group was similar to that observed in the RDN-CKD study (Fig. 5 right side), while eGFR was not adversely affected [52]. Thus, in two independent study cohorts and study investigators, RDN has been documented to reduce in particular diastolic BP under both ambulatory and office BP conditions.

### Nephroprotective effects

Data from the Global SYMPLICITY Registry confirm that eGFR decline per year was not significantly different between the CKD and non-CKD groups 1 year after RDN [45]. This finding was confirmed when analyzing only those subjects with both baseline and 36-month BP data [45]. This result was again confirmed when the Global SYMPLICITY Registry DEFINE was reanalyzed in a larger number of patients, which also separated CKD stages 3a and 3b. After initial changes from baseline to 6 months that were related to the regression to the mean, the observed decline per year was similar to the age-dependent decline per year, i.e. the progressive loss of renal function seemed to be stopped in patients with CKD, but these data are clearly based on nonrandomized, observational data from a worldwide registry [51] and should be interpreted with caution.

Based on single-center observational studies, a number of independent research groups have shown evidence of benefit on eGFR when compared with the historical decline of eGFR in each individual patient. Our own analysis of 27 patients with uncontrolled resistant hypertension and stage 3 CKD (eGFR 30–59 mL/min/1.73 m^2^) confirmed stabilization of eGFR 12 months after RDN compared with the progressive eGFR decline during the 3 years prior to RDN [53]. The same study showed a significant reduction in albuminuria after RDN in patients with resistant hypertension and elevated UACR [53]. Similar results were reported in a small study of 15 patients with CKD [54]. Another observational study showed stabilization of eGFR for up to 24 months after RDN in 46 patients with stage 3 CKD [55]. A study of 30 patients with mild to moderate CKD (mean eGFR 61.9 ± 23.9 mL/min/1.73 m^2^) and refractory hypertension [47] found that eGFR actually increased from baseline after RDN, and the difference remained significant at all time points through the end of follow-up at 24 months [47]. These results are encouraging, but are far from definitive proof that RDN attenuated the decline in renal function in patients with CKD.

Intensive research is ongoing in this area. The safety of RDN in patients with CKD has been further emphasized in 48 patients with treatment-resistant hypertension, which found no changes in the CKD progression marker CKD273 24 months after RDN in patients with eGFR ≥45 mL/min/1.73 m^2^ [56]. The RDN ADPKD trial is currently evaluating the safety and reduction in BP and renal outcome parameters (magnetic resonance imaging cyst growth and measured GFR) of ultrasound-based RDN in patients with autosomal dominant polycystic kidney disease with a 3-year follow-up (clingov: NCT05460169). A similar study to the RDN-CKD study is currently being conducted in Greece.

## RDN IN PATIENTS WITH ESRD

Promising data on the procedure in ESRD patients with resistant hypertension have been reported in small scale pilot studies [57]. A single patient case report [58] and two case series from different RDN sites involving a total of 18 patients reported on the safety and efficacy of RDN in ESRD patients on chronic hemodialysis [24, 59].

An initial safety and proof-of-concept study in patients with ESRD and BP demonstrated that RDN may be safe and effective in reducing BP [24]. Compared with baseline, office systolic BP was significantly reduced at 3, 6 and 12 months after RDN in the nine treated patients (from 166 ± 16.0 to 148 ± 11, 150 ± 14, and 138 ± 17 mmHg, respectively), whereas no change was observed in the three untreated patients [24]. Two patients underwent repeat assessment of sympathetic nerve activity and demonstrated a significant reduction in sympathetic nerve activity [24].

RDN was also successfully performed without complications in six patients with ESRD in our case series. There was a significant reduction in 24-h ambulatory BP of 20 ± 17/15 ± 12 mmHg 6 months after RDN and no change in antihypertensive medication [59]. Special care was taken to avoid any confounding influence of hemodialysis parameters during the study period. Further evidence comes from a randomized controlled trial in patients with ESRD, long-term hemodialysis and treatment-resistant hypertension [60]. The study compared the BP-lowering effect up to 1 year after RDN (12 patients) with the BP effect of medical treatment (12 patients) [60]. No significant periprocedural complications of RDN were observed. Office systolic and diastolic BP and 24-h ambulatory BP showed early and sustained reductions after RDN (24-h ambulatory systolic BP: baseline: 175 ± 11 mmHg, 1 month after RDN: 163 ± 20, 6 months after RDN: 148 ± 10, 12 months after RDN: 149 ± 17 mmHg) [60]. The average number of antihypertensive medications administered did not change during the study [60].

There are very few data on RDN in renal transplant recipients. RDN may be an interesting treatment option in this patient population, as BP elevation associated with renal transplantation is common and often exaggerated after successful transplantation due to immunosuppression. A small randomized trial provided preliminary evidence of the safety and efficacy of RDN in renal transplant recipients [61].

## FUTURE PERSPECTIVES

In the next few years, we face several challenges. First, should there be a sham group? The clinical consensus statement of the ESC Council on Hypertension and the European Association of Percutaneous Cardiovascular Interventions (EAPCI) from 2023 discusses the advantages and disadvantages of a sham-controlled clinical trial design for future RDN trials [49]. For patients with CKD, we should consider that we now have evidence from the Global SYMPLICITY Registry [38], the Erlangen Registry [50] and smaller studies [53] suggesting that RDN is equally safe and effective in its BP-lowering capacity in CKD compared to non-CKD patients. Considering this together with the European guideline recommendations for RDN in patients with eGFR >40 mL/min/1.73 m^2^ [20, 21] and the FDA approval of the ultrasound-based and radiofrequency-based RDN systems, we are faced with the ethical question of whether we can and should use a sham-controlled design in future randomized clinical RDN trials in CKD patients. The consensus statement (written 2 years ago with less available information) concluded that the number of patients assigned to a sham RDN procedure should be minimized as much as possible [49]. Therefore, alternative trial designs are needed. One possible alternative clinical trial design has been used in the RDN ADPKD trial in patients with autosomal dominant polycystic kidney disease (clingov: NCT05460169). While one group is treated with RDN within 1 month of enrolment (immediate group), a second group (delayed group) is treated with RDN 3 months later. However, unpublished data show that the delayed group did not show a reduction in BP during the first 3 months after randomization. In addition, no additional confounding factors were observed in the delayed group. Thus, the setting seems to be applicable and appears to adequately represent a control group (clingov: NCT05460169).

Second, what efficacy parameters should we include in the study design? It would certainly be nice to obtain hard outcome data on RDN, but for several reasons this seems impossible. One point is that, from an ethical point of view, patients in the sham group cannot be maintained at high BP levels for >3 months or at maximum 6 months without additional treatment. In addition, the known confounding factors (change of medication, drug adherence) are exacerbated during the longer follow-up periods after RDN. The use of win-ratio analysis certainly may help to estimate the effect of RDN on BP when medication changes occur. Undoubtfully, measurements of eGFR slope before and after RDN should be included as a renal endpoint in CKD patients.

Third, office BP measurements are necessary but not sufficient to assess the pressure load on the cardiovascular and renal systems. The use of 24-h ambulatory BP and home BP measurements covering the hemodynamic pressure load over a full week seem to be advisable tools for future trials.

Fourth, the high prevalence of non-adherence in the hypertensive CKD population, especially in those patients with treatment-resistant hypertension [62], should make it mandatory to include adherence measurements and subsequent sensitivity analysis of the entire adherent population in clinical trials.

## CONCLUSION

Safety and efficacy data from small clinical trials and observational studies did not observe a safety signal, thereby providing an important information to consider RDN in patients with CKD. Clinical trials and observational studies also found clinically meaningful reductions in systolic and diastolic BP over 3 years after RDN. Thus, RDN appears to be a promising additional treatment option for patients with uncontrolled hypertension and CKD. However, further randomized controlled data are needed to support these findings, especially in patients with advanced CKD.

## Data Availability

The data underlying this article will be shared on reasonable request to the corresponding author.
